# Inclusion of information technology-based assessments of health-related quality of life in routine oncology practice in Uruguay

**DOI:** 10.1186/s41687-022-00458-7

**Published:** 2022-06-13

**Authors:** Cecilia Castillo, Ana Laura Alfonso, Juan J. Dapueto, Natalia Camejo, Martín Silva

**Affiliations:** 1 Department of Clínical Oncology, Facultad de Medicina - Universidad de La República, Hospital de Clínicas “Dr. Manual Quintela”. Av. Italia 2870, Planta Baja, 11600 Montevideo, Uruguay; 2 Department of Medical Psychology, Facultad de Medicina - Universidad de La República, Hospital de Clínicas “Dr. Manual Quintela”. Av. Italia 2870, piso 15, 11600 Montevideo, Uruguay; 3 Humana IT, Enrique Muñoz 887, 11300 Montevideo, Uruguay

**Keywords:** Quality of life, Patient reported outcomes, Clinical practice, Oncology, Uruguay

## Abstract

**Background:**

Previous research has shown that the inclusion of patient-reported outcomes measures in the patient’s visit to the oncologists might improve the quality of global health care. The aim of the study was to assess the feasibility, acceptance, and utility perceived by patients and oncologists of health-related quality of life (HRQL) assessments obtained prior to clinical visits, and to evaluate if this has an impact on patient’s well-being in a sample of Spanish-speaking patients from Uruguay.

**Methods:**

Patients assisted regularly in the Oncology Clinic were randomized into two groups: an intervention group that completed a set of questionnaires (European Organisation for Research and Treatment of Cancer-Quality of Life Questionnaire-C30 (EORTC QLQ-C30) and Hospital Anxiety and Depression Scale using a touch screen device and a control group that did not respond to these questionnaires. At 2 months, the responses of all the participants to the Functional Assessment of Cancer Therapy-General (FACT-G) were collected over a telephone to determine whether there were differences in the HRQL between the intervention and control groups. The graphed scores of the intervention group were included in the clinical history of the patient during consultation. Patients and physicians completed the questionnaires on the usefulness of these measurements.

**Results:**

In total, 58 patients participated in this study: 36 in the intervention group and 22 in the control group; 65% of the participants were female, and median age was 59 years (18–79). Regarding patients, 97% found the questionnaires easy to complete and thought that they included important questions. As for oncologists, 68.8% used the information and 87.5% found it useful for the consultation. There were no significant differences in the FACT-G scores between the intervention and control groups.

**Conclusions:**

The routine HRQL assessments using an electronic device prior to the consultations were positively valued by almost all patients and physicians. This could significantly contribute to a better understanding of the patient's overall problems during consultation. These results confirm the benefits of integrating the patient’s self-reported quality of life outcomes into consultations.

**Supplementary Information:**

The online version contains supplementary material available at 10.1186/s41687-022-00458-7.

## Background

Health related quality of life (HRQL) research in oncology has rapidly evolved in the last decades from the initial efforts to develop reliable and well validated measures of symptom burden, well-being, and quality of life, to the inclusion of patient-reported outcomes measures (PROs) in clinical trials, and more recently to the application of PROs in daily clinical practice.

Several studies demonstrated that PROs obtained in an individual level can be used in the patient’s visit to the oncologists to facilitate the detection of physical or psychosocial problems, to monitor disease progression, treatment effectiveness and side effects, to improve patient-doctor communication and to improve the quality of global health care [1–3]. Self-reports obtained prior to the consultation may increase the number of topics discussed as well as the quality of global health care even when it may not have a demonstrated impact on the decision-making process [4–7].

Therefore, the inclusion of standardized questionnaires on the quality of life in clinical practice can help health professionals become acquainted with the assessment of functional and psychosocial problems, thereby contributing to the better management of these issues. Monitoring adverse effects has been highlighted as another source of valuable information from patient reports that can improve the treatment adequacy [7].

Some critical aspects need to be solved when including PROs in daily clinical practice such as the development of computer-assisted data collecting procedures, including the use of portable devices with audio-visual testing available for patients with visual or reading disabilities, real-time data capture and processing, generation of graphic outputs easily comprehensible to physicians, that can be printed and included in the medical chart during the short period of time that spans from the patient arrival to the clinic to the moment he or she enters the doctor’s office [2].

The use of computerized patient data collection methods has enabled real-time HRQL measurements and presentation of the results to clinicians, which is a feasible approach in busy daily clinical practice [1, 3, 4, 6]. The patient feedback on the use of these methods has revealed a high degree of acceptance [7]. Chen et al., conducted a review on the implementation of PRO measures in routine oncology practice based on the identification of a set of outcomes [8]. They concluded that there is growing evidence supporting the routine collection of PRO to enable better and patient-centered care, patient-provider communication and improves patient satisfaction, improves the monitoring of treatment response and the detection of the unrecognised problems. However, the evidence was weak for its impact on changes to patient management and improved health outcomes and non-existent for changes to patient health behavior, strong and effective quality improvement, increased transparency, accountability, public reporting and better health care system performance. They suggested that these evidence gaps require further research. In addition to the well-accepted PROs, the application of computer adapted testing, the acceptance of information technology by patients and health professionals, the real-time and routinely collected PROs might enable a more patient-centered health care system.

Another important research gap, as shown in the review, is that the 27 articles identified had been conducted in referral cancer centers in North America, Europe, or Australia, in populations with high socioeconomic status. Therefore, it is very relevant to know how electronic data collection procedures behave in a sample of low-income patients treated at a public university hospital in a South American country. It is highly relevant to include audiovisual evidence of PRO in populations historically excluded from HRQL studies, who are the ones who will experience the impact of cancer and treatments on their general well-being to a greater extent [9].

The aim of this research was to study, as a primary outcome, the feasibility, acceptance, and utility perceived by patients and oncologists of the inclusion of the patient’s assessments of their HRQL obtained immediately prior to the routine clinical visit, and as a secondary outcome, to evaluate if this information provided to the oncologist may have an impact on the patient’s well-being and the satisfaction with care two months after the first assessment.

It was hypothesized that the application and inclusion of serial HRQL measurements in the oncology clinical practice would be well accepted and considered useful by both patients and oncologists. Secondly, it could cause positive changes in the quality of care, leading to greater patient satisfaction with medical care as well as greater adequacy of medical decisions, better control of symptoms, and better perception of quality of life.

Therefore, we intended to provide a tool to our oncologists for the convenient and reliable measurement of the quality of life of their patients that can be incorporated into their clinical evaluations.

## Method

We designed a longitudinal study following the methodology and recommendations proposed by Galina Velikova [4, 6] modified to make it feasible for our medical setting. It included the randomization of patients in two groups, the intervention group consisting of the completion of quality of life questionnaires and providing feedback to doctors as part of medical notes, and a control group that will not complete the quality of life questionnaires in the clinic.

All patients assisted at the Clinical Oncology Department of the Hospital de Clínicas – Universidad de la República, Uruguay between March and July 2017 were invited to participate. The eligibility criteria were patients aged 18 years or older, those with a diagnosis of cancer that could be treated systemically (chemotherapy, hormone therapy, or biological therapies) with a life expectancy of at least 6 months, and those who were starting such treatment or had started it in the last 3 months. Patients with a concurrent medical disease, with cognitive deficits, dementia or with a severe persistent mental illness were excluded.

Sample size calculation was based on a minimal important difference in the FACT-G total score of 10 points (range 0–108 points), an expected standard deviation in each arm of 9 points, a sample size ratio of 1, with 95% confidence interval, and 80% statistical power. Estimated sample size was 41 patients for each study arm.

### Measures

The following instruments were included in the initial clinical assessment using an electronic device.

The **European Organisation for Research and Treatment of Cancer-Quality of Life Questionnaire-C30 (EORTC QLQ-C30)** [10, 11] is the HRQL measurement instrument of the European Organization for Research and Treatment of Cancer considered reliable and valid in various linguistic and cultural contexts [12]. It includes 30 items divided into five functional scales (physical, role, emotional, social, and cognitive functioning), three symptom scales (fatigue, pain, and nausea/vomiting), overall health/quality of life scale, and several items assessing additional symptoms (dyspnea, loss of appetite, insomnia, etc.) and the financial impact. The measurement scale ranges from 0 to 100. For the five functional scales and overall quality of life scale, a higher score represents a better level of functioning, whereas for the symptom scales, a higher score indicates a higher level of symptoms.

The **Hospital Anxiety and Depression Scale (HADS)** [13–15] is a brief and reliable self-reported measure of anxiety and depression in hospitalized and ambulatory medical patients, also used in primary care and research, consisting of seven items for anxiety (HADS-A) and seven for depression (HADS-D). The Spanish version of the HADS is available from the publisher (GL Assessement, www.gl-assessment.co.uk).

During the follow-up, The **Functional Assessment of Cancer Therapy Questionnaire Spanish Version 4 (FACT-G)** was completed through a telephone interview. The FACT-G [16, 17] is comprised of 27 items measuring four dimensions of HRQL using 5-point Likert-type response categories ranging from 0 = 'not at all' to 4 = 'very much’: physical well-being (PWB, 7 items, range 0–28 points), social and family well-being (SFWB, 7 items, range 0–28 points), emotional well-being (EWB, 6 items, range 0–24 points), and functional well-being (FWB, 7 items, range 0–28 points). It provides a FACT-G Total that is the summation of the subscale scores (range 0–108), and a Total Outcome Index score (TOI) that summarizes the PWB and FWB scores. The FACT-G was validated in Uruguay [18]. Higher total and subscale scores represent better quality of life and well-being.

The **Visit Specific Doctor Questionnaire and End of Study Doctor Questionnaire **(VSD1), a self-reported questionnaire developed by Prof. Galina Velikova from the University of Leeds School of Medicine (personal communication) was used to evaluate the oncologists' perception of the clinical utility of having the patient’s self-reported HRQL information during consultation. It assesses the oncologists’ perception on the utility of the testing in the following areas, providing overall assessment of the patient, providing additional information, confirming knowledge of patient’s problems, identifying issues/problems to be discussed, starting/stopping/changing symptomatic drugs, ordering tests, starting/stopping chemotherapy, referral to psychologist, counselling about lifestyle. All the members of the Oncology Department were invited to participate. The physicians worked in 8 teams with a total of 20 physicians including residents. Different physicians attended the patients during the follow up, so the patients saw different physicians over time.

The battery included an ad-hoc test on patients' satisfaction and potential benefits of the procedure, considering how easy or difficult it was to complete, how time consuming they considered the procedure, whether they were willing to complete the tests at each visit, if the questions were relevant, if they would prefer to answer using the touch screen device or to have the questions read out in an interview. It also included a list of clinical and sociodemographic data.

### Software development

The research design made necessary to develop two software applications. One uses an iPad^®^ device that captures the information from patients (audiovisual computer-assisted testing software or AVCT) and send it through the Internet, and a web-based application. Screenshots of how the questions looked from the patient’s perspective are shown as Additional File 2, Additional File 3 and Additional File 4. The graphical presentation of results to the clinicians are shown in detail as Additional File 1.

The software also includes optional audio recordings to be used by people with low reading skills or visual limitations. The web application used PHP5 as programming language, PostgreSQL for database, and Apache2 as web server. This application receives the information from patients and doctors, calculate scores, generate graphic reports on PDF format of the several indicator or dimensions of the questionnaires.

### Procedure

To ensure variability, data collection was performed on two different days of the week. The patients approached were cared for by different treatment teams that included members of the teaching staff and residents. The patients were randomized into two groups: an intervention group that completed a set of HRQL questionnaires using a touch-screen device with a telephone follow-up and a control group that did not respond to any questionnaires at the start of the study but only to a telephonic follow-up survey.

The measurements for the patients in the intervention group were conducted in the waiting room before the consultation with the oncologist on two different occasions: at the time of inclusion in the study (T1) and at 2 months (T2). The EORTC QLQ C-30 and the HADS were responded at every time.

The follow-up measurement was conducted over the telephone for both groups using the FACT-G questionnaire. It was conducted within 48 h after the measurement at T2 for the intervention group and 2 months after signing the informed consent form for the control group.

### Statistical analyses

A descriptive analysis was performed to calculate the frequencies for the qualitative variables and measures of dispersion for the continuous variables. The statistical t-test for paired samples was applied to study the differences between the two measurement times in the intervention group. The difference in the means of the measurements of the quality of life between the intervention and control groups (FACT-G) was studied using the t-test for independent samples. All analyses were evaluated at a significance level of 0.05.

## Results

**Fig. 1:**
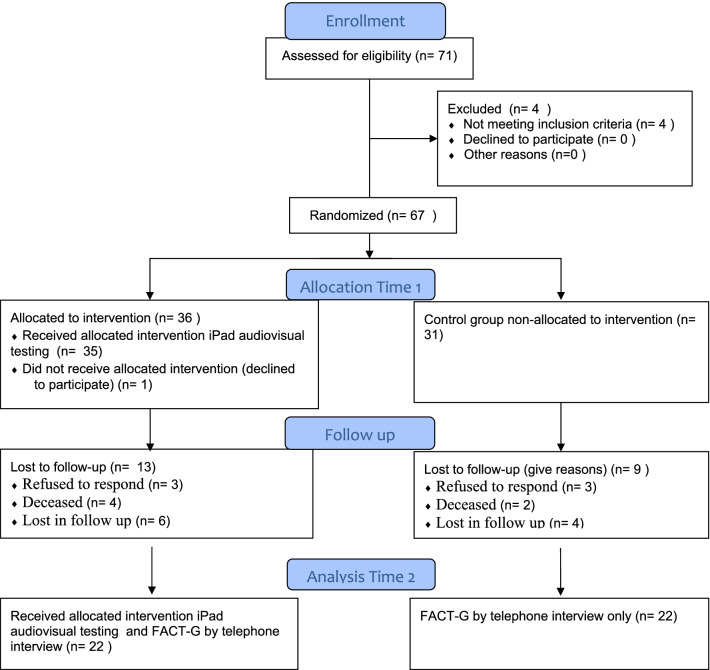
Sample flow chart

The number of patients approached and the description of the sample at baseline and follow-up are shown in Fig. 1. In total, 58 patients participated in this study: 36 in the intervention group at the time of recruitment and 22 in the control group at the follow up (Time 2). Of the total participants, 65.5% (n = 38) were female, with mean age of 59 years, range: 18–79. The sociodemographic and clinical characteristics of the sample are presented in Table 1.

**Table 1:** Sociodemographic and clinical features

	Intervention group (n = 36)	Control group (n = 22)	Total (n = 58)
Age: mean (range)	58 years (28–79)	60 years (18–74) (%)	59 years (18–79) (%)
Gender	%	%	%
Female	66.7	63.6	65.5
Male	33.3	36.4	34.5
Marital status	%	%	%
Married/unmarried couple	41.7	50	44.8
Divorced /separated/widow	27.8	22.6	25.8
Single	27.8	18.2	24.1
Household composition	%	%	%
No data	2.8	9.1	5.2
Living alone	11.1	13.6	12.1
Living with relatives or others	86.1	77.2	82.7
No data	2.8	9.1	5.2
Occupation	%	%	%
Full time or partial time job	22.2	13.6	18.9
Retired/pension/medical leave	44.4	50	46.5
Unemployment insurance/ looking for a job	16.6	22.7	18.9
Student	2.8	0	1.7
No data	13. 8	13.6	13.7
Profess a religion	%	%	%
Yes	69.4	40.9	58.6
No	30.6	59.1	41.4
Type of cancer	%	%	%
Breast and gynecological	47.2	54.5	50
Lung	13.8	9.1	12.1
Urologic	2.7	9.1	5.2
Digestive	33.3	18.1	27.5
Other	12.7	9.1	5.2
Type of treatment	%	%	%
CT or CT + RT	83.3	77.2	81
Biological therapy	5.5	4.5	5.1
CT + Biological therapy	8.3	4.5	7.9
Hormone therapy	0	9	3.4
Other	2.8	4.5	3.4
Treatment intention	%	%	%
Adjuvant/neoadjuvant	55.5	54.5	55.1
Definitive CT/RT or Hormonal/RT	11.1	13.6	12.1
Paliative	33.3	31.8	32.7

*CT* Chemotherapy, *RT* Radiotherapy

Regarding the utility and acceptance of the use of the touch screen by patients at T1, 97.1% of the patients considered it “easy” and “very easy” and that the questions were important, 48.6% supported the use of the touch screen, 31.4% responded that the methods were “the same,” and 20% preferred having a person read the questions to them.

The VSD1 questionnaire was completed by 16 physicians (oncologists and residents) at T1 and T2. The responses are presented in Table 2. Notably, 75% of the physicians did not know or only partly knew the patient at the time of the first interview, 68.8% used the questionnaire information, and 87.5% responded that they found the information useful during the consultation.

**Table 2:** Oncologists’ responses to the VSD1 questionnaire

	Time 1 (%)	Time 2 (%)
*How well did you know this patient from before?*		
Never met him/her before or a little bit	75	43.8
Moderately or very well	25	56.2
*Did you look at the QL results before the consultation?*		
Yes. very much or quit a bit	68.8	68.8
Somewhat or a little	31.2	31.2
No	0	0
*Did you use the QL information in the clinic discussion?*		
Yes. very much or quite a bit	87.5	68.7
Somewhat or a little	12.5	31.3
No	0	0
*How was the information useful for the consultation?*		
Providing overall assessment of the patient	93.7	75
Providing additional information	62.5	31.3
Confirming your knowledge of patient’s problems	37.5	31.3
Identifying issues/problems to be discussed	31.3	25
Starting. stopping. changing symptomatic drugs	18.7	6.3
Ordering tests	6.3	6.3
Starting. stopping chemotherapy	12.5	0
Referral to psychologist	31.3	25
Counselling about lifestyle	12.5	0
Other	12.5	18.7
*Do you feel that the QL data had any impact on consultation length?*		
Yes. lengthened	31.3	25.4
Yes, shortened or made no difference	68.7	74.6
Not sure	0	0

*VSD1* Visit Specific Doctor Questionnaire and End of Study Doctor Questionnaire

When asked in what aspects was the survey useful, the best ratings were obtained by: providing overall assessment of the patient (93%), and providing additional information (62.5%); the best rated contribution to management was the request for mental health consultation by psychologist (31.3%) followed by stopping or changing symptomatic drugs (18.7%) (Table 2). Almost 69% considered that the survey did not prolonged the interview or even helped to shortened it. Comparing the first and second evaluation, the survey was still considered useful by the majority of doctors (68.8%) and provided significant clinical information for 75% of them.

The scores of the questionnaires are presented in Tables 3 and 4. No significant differences were observed in the EORTC QLQ-C30 Global Health Index, subscale and symptom scores at T1 versus T2. Similar results were observed in the HADS Anxiety and Depression mean scores.

**Table 3:** Comparison of EORTC QLQ-30 scores and HADS scores at Time 1 and Time 2

	Time 1	Time 2	Time 2 vs 1		
	Mean	SD	Mean	SD	p
*EORTC QLQ-30 Scores*					
Global Health Index*	68.81	25.35	65.53	16.12	ns
Emotional functioning*	70.48	23.51	72.73	24.16	ns
Performing social roles*	78.57	25.98	80.30	26.54	ns
Physical functioning*	82.67	17.65	82.74	17.90	ns
Cognitive functioning*	84.76	20.76	86.35	14.21	ns
Social functioning*	83.34	19.79	81.05	24.28	ns
Financial problems*	30.47	30.65	31.81	24.08	ns
Fatigue**	34.91	24.28	31.81	25.78	ns
Nausea and vomiting**	6.19	12.83	8.33	14.31	ns
Dyspnoea**	17.14	31.69	19.69	31.97	ns
Anorexia**	15.23	28.40	12.11	19.36	ns
Insomnia**	28.56	30.41	27.27	30.24	ns
Constipation**	18.09	30.62	21.21	26.32	ns
Diarrhea**	10.47	23.94	16.67	30.44	ns
HADS Scores					
Depression Scale***	377	301	473	383	ns
Anxiety Scale***	626	391	495	405	ns

*EORTC QLQ-30* European Organisation for Research and Treatment of Cancer-Quality of Life Questionnaire-C30, ns non-statistically significant

^*^Score range 0–100, higher scores indicating better performance

^**^Score range 0–100, higher scores indicating more severe symptom

^***^Score range 0–21, higher scores indicating more sever symptom

**Table 4:** Differences in the FACT-G scores between the Intervention and Control groups at Time 2

FACT-G Subscales		Intervention group % (N = 22)		Control group % (N = 22)		
	Score range	Mean	SD	Mean	SD	*p*
Physical well -being	0–28	23.14	4.37	20.86	5.22	0.1
Social well -being	0–28	18.99	4.98	18.51	4.59	0.7
Emotional well- being	0–24	16.40	4.49	15.91	3.71	0.7
Functional well-being	0–28	17.91	4.16	17.31	6.00	0.7
FACT- G Total Score	0–108	76.44	13.51	72.60	15.67	0.4

*FACT–G*, Functional Assessmement of Cancer Therapy–General Questionnaire

Higher scores indicate better performance

The results of the FACT-G questionnaire are presented in Table 4. Higher scores in the FACT-G Subscale and Total Scores were observed in the ratings of the intervention group compared with the controls, but these differences were not statistically significant.

## Discussion

Several studies have shown that an electronic data collection method is easy, fast, reliable, and acceptable to patients, which are all important factors for integrating the measurements of the quality of life into daily oncology practice [8, 19–21]. Most, if not all, of these studies have conducted in references centers of high income countries. To our knowledge this is the first study of its kind in a middle-income South-American country, addressing Spanish-speaking patients of a public hospital.

In our study, the patients showed high compliance with the assessment of the HRQL aspects during routine medical consultations, and the patients appreciated that the physicians consider such assessments. More than 95% of the patients considered that the questionnaires included important questions and were willing to complete them prior to each medical consultation. Moreover, there was good acceptance for completing the questionnaires using a touch screen, which the patients found easy and convenient. However, 20% of the patients still preferred having another person read the questions to them. This could be attributed to the high percentage of elderly people in our population with no previous experience of using this type of portable equipment.

In general, the physicians showed very good acceptance for incorporating the HRQL into their daily clinical practice. The use of the HRQL information contributed to a better understanding of the patients and their problems, supporting decision-making in the consultation for treatment changes, and referral to mental health specialists. The time of consultation was not measured in our study, only the physician’s perception on the length of the consultation was registered. However, the majority of the oncologists perceived that having the graphical output of the measures did not prolonged the consultation time, specially at T2. For these doctors having PRO results might improve the effectiveness and efficiency of the consultation. However, a substantial proportion of oncologists reported it lengthened the encounter (31.3% at T1 and 25.4% at T2). This is a relevant issue since the acceptance by physicians is a key aspect for the implementation of the PROs, and probably requires more training for the rapid reading and interpretation of the results in easy-to-use graphic formats and with clear criteria on the clinical significance of the scores.

The results of the physician questionnaires showed that the HRQL information during the consultation was more useful in T1 than in T2. Although physicians knew their patients better in the second interview, previous studies also showed a positive impact on the doctor-patient relationship [3, 6, 22].

Regarding the second hypothesis, the intervention based on the HRQL assessments included in daily clinical practice did not have a positive impact on the patients’ quality of life as measured by the FACT-G questionnaire two months after the first assessment. However, it is expected that changes in the quality of life appear later over the course of treatment, especially after completing the active treatment; hence, it would be necessary to extend the follow-up time and conduct new assessments.

As proposed by different HRQL researchers worldwide, future efforts should be directed at improving the flexibility and accuracy of the HRQL assessments and at linking patients' HRQL scores to specific treatment and care strategies. In this sense, some centers have described their experiences on how to integrate the quality of life outcomes that are regularly self-reported by oncology patients through a web application or telephone system [23–25]. Furthermore, a randomized study of patients with disseminated disease undergoing chemotherapy suggests a positive impact on patient survival upon integration of such outcomes [26]. Despite the potential benefits, there are several challenges for integrating this information into clinical practice as with any other change in the existing clinical processes [27].

This study has several limitations, such as the small sample size of a single cancer center, diversity of patients in terms of diagnosis and stage, and short follow-up period, and infrequent measurements. The sample size was not reached due to difficulties in the logistics of the data collection procedure during the planned time of the investigation, such as a slower accrual of patients than anticipated, that exceeded the possibilities of control of the research team. We agree that this is a limitation of the study, as it could have an impact on the significance of the results at Time 2. However, we believe that these results provide clear guidance on the design and implementation of future studies in this area. As the intervention measurements were made only at two points two months apart, and not at each chemotherapy visit that is expected to be every three to four weeks. Since the outcome measure, FACT-G, was completed only at time 2, two months after study entry, and there is no baseline data, it is possible that the two groups of patients had different baseline measures, despite randomization. This is one potential explanation to the lack of effect on patient well-being.

However, it should be noted that as a result of our study, a computer application was developed consisting of a program for computerized audiovisual testing using a touch-sensitive screen, which included validated instruments for measuring the quality of life. This program can be implemented for clinical evaluation in other cancer centers in the country, as well as in clinical research that includes the patient's perspective as an additional outcome variable.

## Conclusions

The inclusion of routine HRQL assessments in daily oncology practice using a touch screen was positively valued by almost all patients and a majority of oncologists. Moreover, it had a favorable impact during the consultations by providing the physician with information about the general problems of the patient, regarding the effect of the oncological disease and its treatments on the physical, functional, psychological and social wellbeing of the patient. Using this measurement procedure on a regular basis may lead to improved therapeutic interventions in our clinical setting as was reported in major western cancer centers.

## Supplementary Information

**Additional file 1.** Graphical presentation of the results

**Additional file 2.** Screenshot of the device

**Additional file 3.** Screenshot of the device

**Additional file 4.** Screenshot of the device

## Data Availability

The datasets used and/or analyzed during the current study are available from the corresponding author on reasonable request.

## Abbreviations

EORTC QLQ-C30: European Organisation for Research and Treatment of Cancer-Quality of Life Questionnaire
FACT G: Functional Assessment of Cancer Therapy Questionnaire Spanish Version 4
HADS: Hospital anxiety and depression
VSD1: Velikova’s Visit Specific Doctor Questionnaire and End of Study Doctor Questionnaire
