# Supplementary material for: Inclusion of information technology-based assessments of health-related quality of life in routine oncology practice in Uruguay
Source: J Patient Rep Outcomes. 2022 Jun 13;6:65. doi: 10.1186/s41687-022-00458-7 (PMC9192877; doi:10.1186/s41687-022-00458-7)

http://cdvhc.humanait.com/seguimiento/index/idp/86

Estudio de calidad de vida de pacientes oncológicos

Resumen - Funcionamiento - 15/04/2015

Paciente: [redacted]

Nota: La linea representa el valor promedio de una población con cáncer a nivel mundial.

Funcionamiento físico

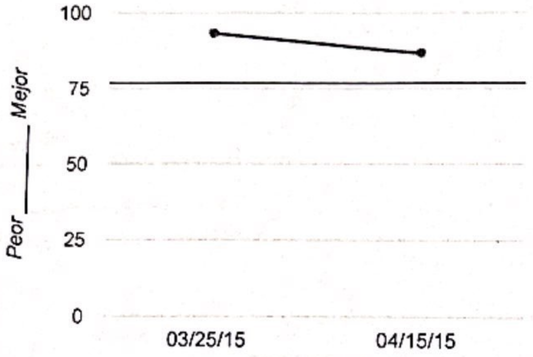

Funcionamiento rol

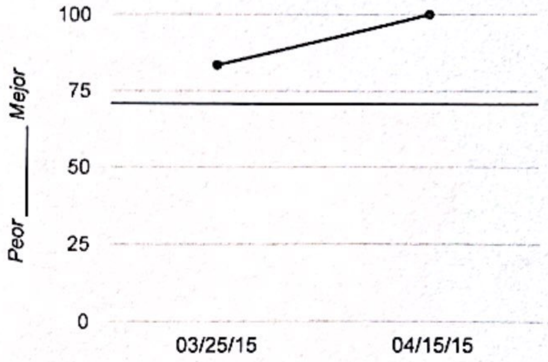

Funcionamiento emocional

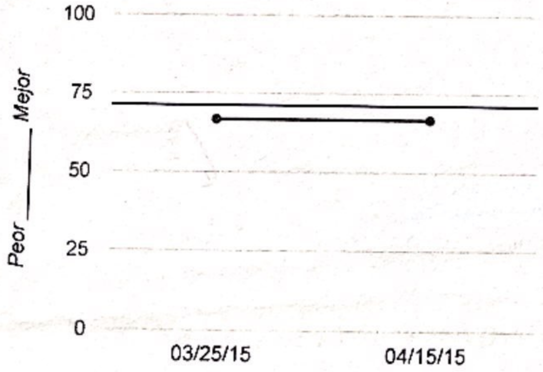

Funcionamiento social

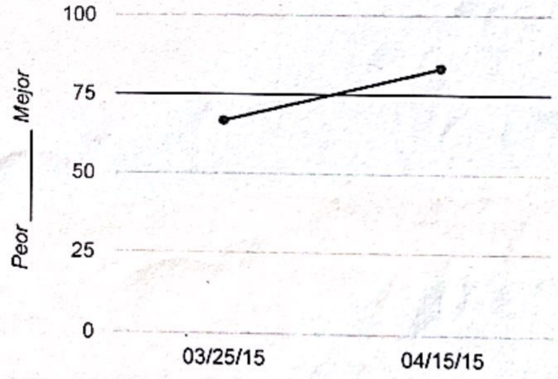

Funcionamiento cognitivo

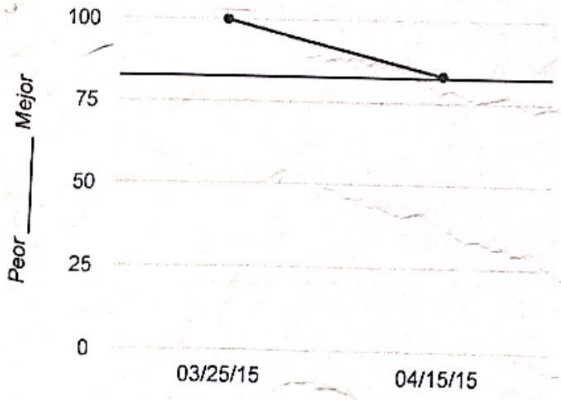

Escala global

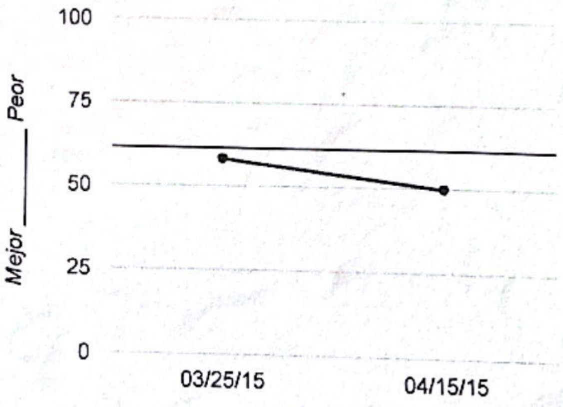

Estudio de calidad de vida de pacientes oncológicos

Resumen - Funcionamiento - 15/04/2015

Fatiga

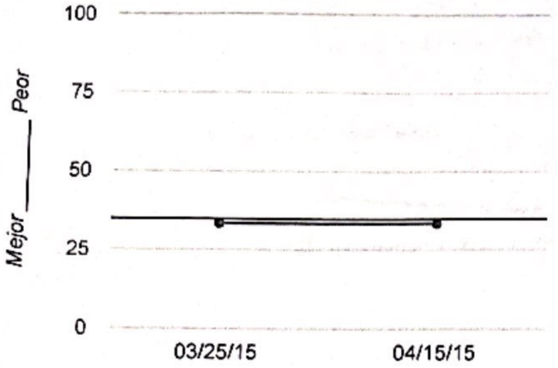

Náuseas y vómitos

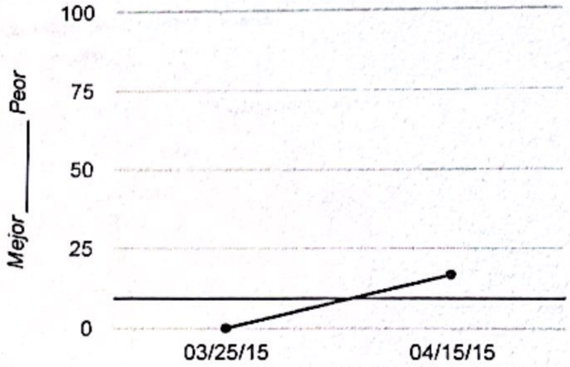

Dolor

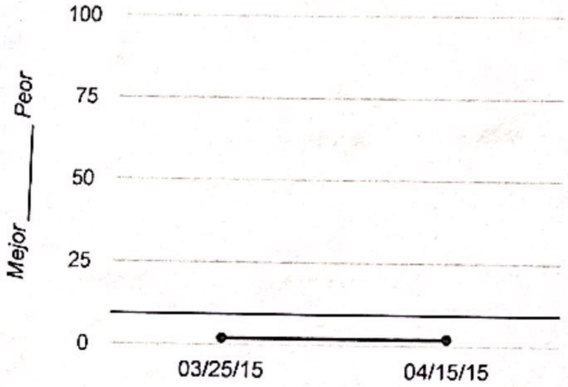

Disnea

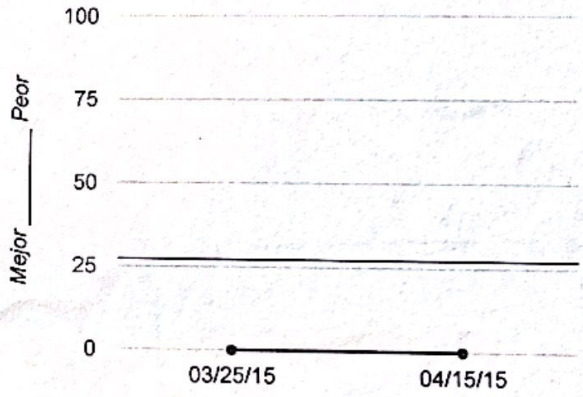

Anorexia

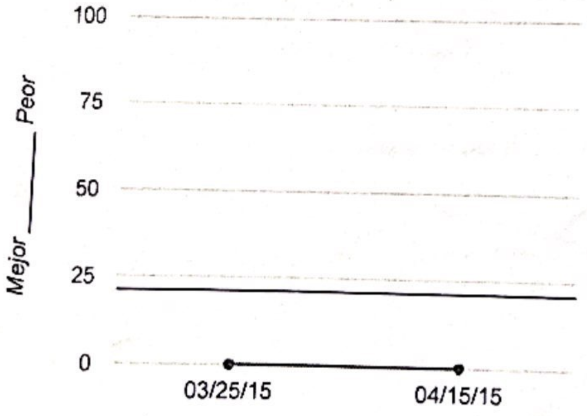

Insomnio

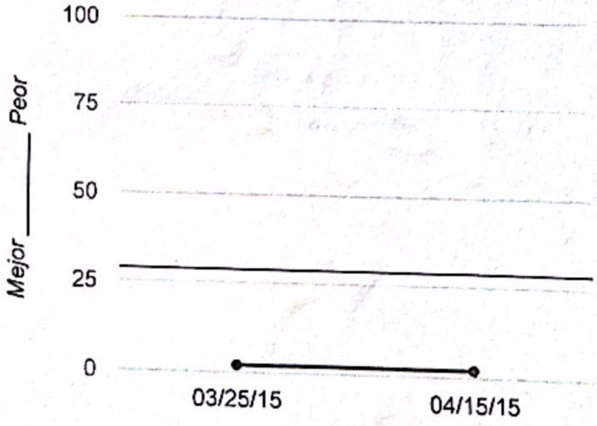

Estudio de calidad de vida de pacientes oncológicos

Resumen - Funcionamiento - 15/04/2015

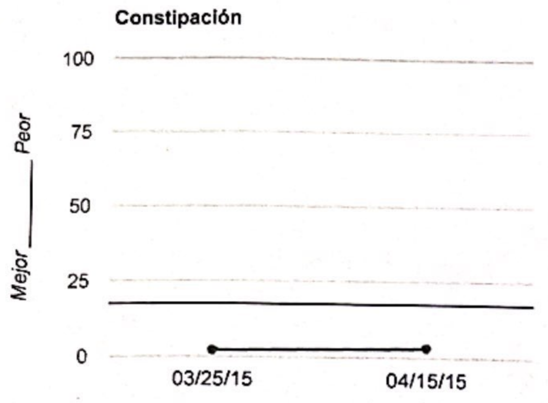

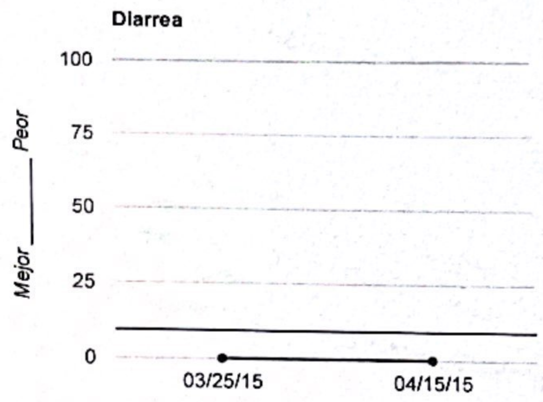

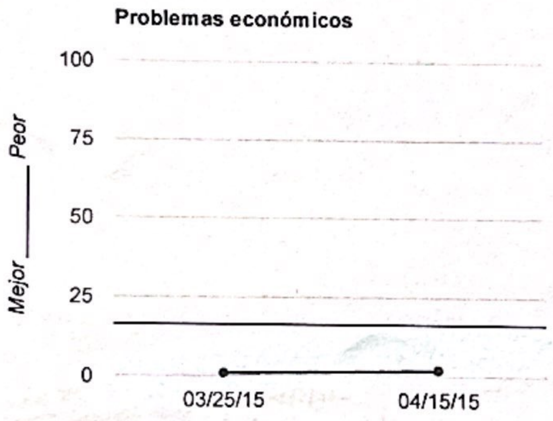

Ansiedad y Depresión (HADS) - 15/04/2015 (1/3)

Nota: El score va del 0 al 21. Score entre 8-11 es borderline, si score >= 11 probable ansiedad o depresión.

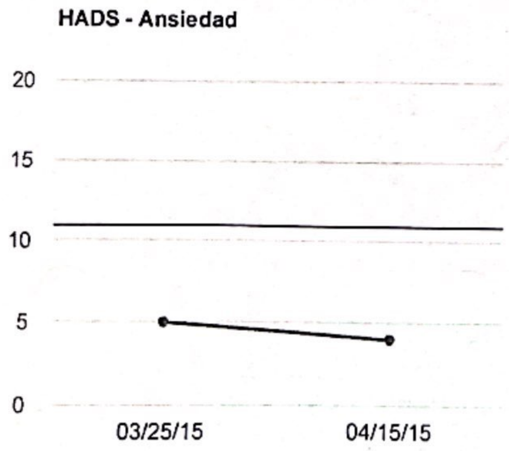

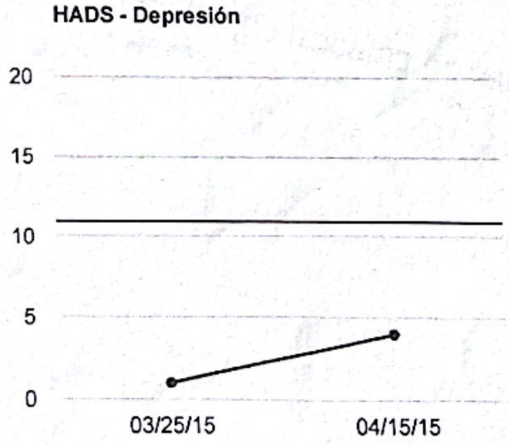

Supplement: Supplementary file 1 — Additional file 1. Graphical presentation of the results [file 41687_2022_458_MOESM1_ESM.pdf]
