# Supplementary figures and images for: Inclusion of information technology-based assessments of health-related quality of life in routine oncology practice in Uruguay
Source: J Patient Rep Outcomes. 2022 Jun 13;6:65. doi: 10.1186/s41687-022-00458-7 (PMC9192877; doi:10.1186/s41687-022-00458-7)

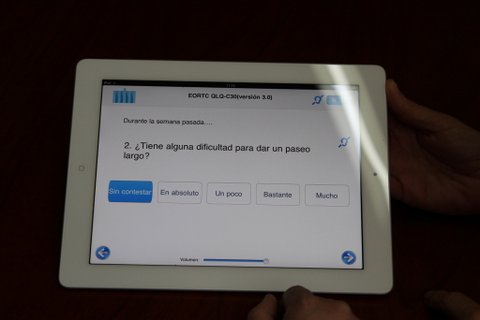

Supplement: Supplementary file 2 — Additional file 2. Screenshot of the device [file 41687_2022_458_MOESM2_ESM.jpg]

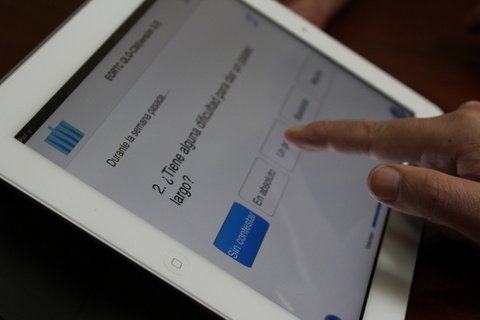

Supplement: Supplementary file 3 — Additional file 3. Screenshot of the device [file 41687_2022_458_MOESM3_ESM.jpg]

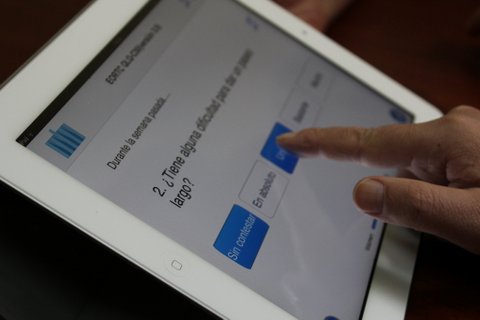

Supplement: Supplementary file 4 — Additional file 4. Screenshot of the device [file 41687_2022_458_MOESM4_ESM.jpg]
